# ECGAssess: A Python-Based Toolbox to Assess ECG Lead Signal Quality

**DOI:** 10.3389/fdgth.2022.847555

**Published:** 2022-05-06

**Authors:** Linus Kramer, Carlo Menon, Mohamed Elgendi

**Affiliations:** ETH Zürich, Biomedical and Mobile Health Technology Lab, Zurich, Switzerland

**Keywords:** data science, digital health, anaesthesia, emergency and critical care, intensive care unit, biomedical engineering

## Abstract

Electrocardiography (ECG) is the method most often used to diagnose cardiovascular diseases. To obtain a high-quality recording, the person conducting an ECG must be a trained expert. When these experts are not available, this important diagnostic tool cannot be used, consequently impacting the quality of healthcare. To avoid this problem, it must be possible for untrained healthcare professionals to record diagnostically useful ECGs so they can send the recordings to experts for diagnosis. The ECGAssess Python-based toolbox developed in this study provides feedback regarding whether ECG signals are of adequate quality. Each lead of the 12-lead recordings was classified as acceptable or unacceptable. This feedback allows people to identify and correct errors in the use of the ECG device. The toolbox classifies the signals according to stationary, heart rate, and signal-to-noise ratio. If the limits of these three criteria are exceeded, this is indicated to the user. To develop and optimize the toolbox, two annotators reviewed a data set of 1,200 ECG leads to assess their quality, and each lead was classified as acceptable or unacceptable. The evaluation of the toolbox was done with a new data set of 4,200 leads, which were annotated the same way. This evaluation shows that the ECGAssess toolbox correctly classified over 94% of the 4,200 ECG leads as either acceptable or unacceptable in comparison to the annotations.

## 1. Introduction

Globally, cardiovascular diseases (CVDs) are the leading cause of death. An estimated 17.9 million people died from CVDs in 2019, representing 32% of all global deaths. Over three-quarters of CVD deaths occur in low- and middle-income countries. It is important to detect CVDs as early as possible to begin management with counseling and medication (1, 2).

Electrocardiography (ECG) is the procedure most often used to diagnose heart diseases. The electrical activity created by the patient's heart is processed by the ECG machine and either printed on special graph paper or digitally recorded. The ECG's popularity is due to its advantages as a non-invasive, inexpensive, and convenient screening tool that is comfortable for patients. Additionally, the procedure of recording ECGs are extremely safe, which further contributes to its popularity.

However, ECGs must be recorded and analyzed by trained experts. Especially in developing countries, where experts are concentrated in urban hospitals, this can lead to a medical undersupply in rural areas. This major problem was targeted by the PhysioNet/Computing in Cardiology Challenge 2011. The goal of this challenge was for participants to develop an efficient algorithm able to run in near-real-time on a mobile device that can provide useful feedback to laypeople in the process of acquiring a diagnostically useful ECG. The software should be able to indicate within a few seconds—while the patient is still present—whether the ECG recording is of adequate quality for interpretation or if another recording should be made. This could technically allow inadequately trained personnel to obtain ECG recordings that can be interpreted without waiting for an expert to determine the quality; it may be difficult to obtain another ECG on another day from a patient who may live far from the clinic (3).

**Figure 1:**
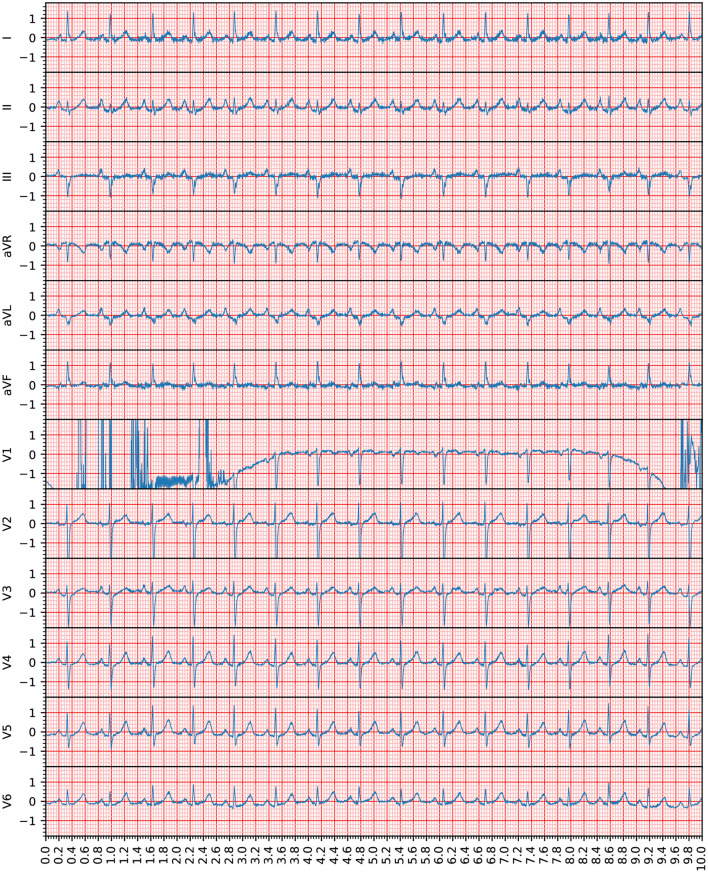
This recording (Recording 1609343) was annotated as being of unacceptable quality in the PhysioNet/Computing in Cardiology Challenge. However, according to the assessment in this study, only Lead V1 was unacceptable because the heartbeats provided by this lead were not clearly recognizable. The voltage (*y* axis) is given in 100 micro volts, the time (*x* axis) is given in seconds. Note that the 12-lead ECG signals are visible in the standard ECG grid to improve the clinical interpretation of the ECG waveforms.

The participants of the PhysioNet/Computing in Cardiology Challenge developed a program that indicates whether an ECG is of sufficient overall quality for a diagnosis, as seen in Figure 1 (4–10). This study goes one step further and provides this feedback for the recording's individual leads. The advantage of this approach is that, in the event of an insufficient recording, it is only necessary to reattach the indicated leads. In this way, high quality signals can be preserved and the correction can be reserved for the poor-quality leads. This innovation saves time and makes the program easier to use.

Because a variety of terms can be used in reference to the signals discussed in this article, the terms must be clearly defined: A 12-lead ECG signal will be referred to as an *ECG recording*, and the signal of one lead in an ECG recording will be referred to as a *signal*.

## 2. Methods

### 2.1. Used Data

The ECG recordings for this study were collected as part of Sana's PhysioNet/Computing in Cardiology Challenge and were provided for free *via* PhysioNet. This data set was used in the PhysioNet/Computing in Cardiology Challenge 2011. The data include 10-s recordings of 12-lead ECGs that were collected using conventional ECG machines. All the leads were simultaneously recorded, have a full diagnostic bandwidth (0.05–100 Hz), and were sampled at 500 Hz with a 16-bit resolution. Nurses, technicians, and volunteers with varying levels of training recorded the ECGs. Because the goal of the PhysioNet/Computing in Cardiology Challenge is to investigate whether laypersons can use software to collect high-quality ECGs reliably; the recordings gathered for the challenge include ECGs taken by volunteers with minimal training.

The publicly available Data Set A consists of 998 ECG recordings, classified into one of two groups. The classifications were made by a group of annotators with different levels of experience in ECG analysis. Group I consists of acceptable ECG recordings, and Group II consists of unacceptable ECG recordings. Approximately 70% of the collected recordings were assigned to Group I and 30% were assigned to Group II. It is important to mention that the categorization into these groups was made based on all 12 leads. The classification did not allow any direct conclusions to be drawn about the quality of individual leads (3).

### 2.2. Annotation

For this project, the ECG recordings were evaluated as individual leads, not as a whole; thus, the above-mentioned division into Group I and Group II could not be used to verify the toolbox. A data set with annotations of acceptable quality and unacceptable quality for each lead was needed. For this purpose, two data sets were created: a training data set with 50 randomly selected ECG recordings from each group (Group I and Group II) and a testing data set with 175 randomly selected ECG recordings from each group. The data sets for both groups each contain the same number of recordings, which should lead to meaningful results.

All the signals from the testing data set were preprocessed using a bandpass Butterworth filter. The recommended bandpass frequency range for detecting QRS complexes is 8–20 Hz (11). The selected filter has a bandpass of 8–20 Hz and stopbands of 0–0.5 and 30–250 Hz (Nyquist frequency). Minimum loss in the stopband is 20 dB; maximum loss in the passband is 0.2 dB.

**Figure 2:**
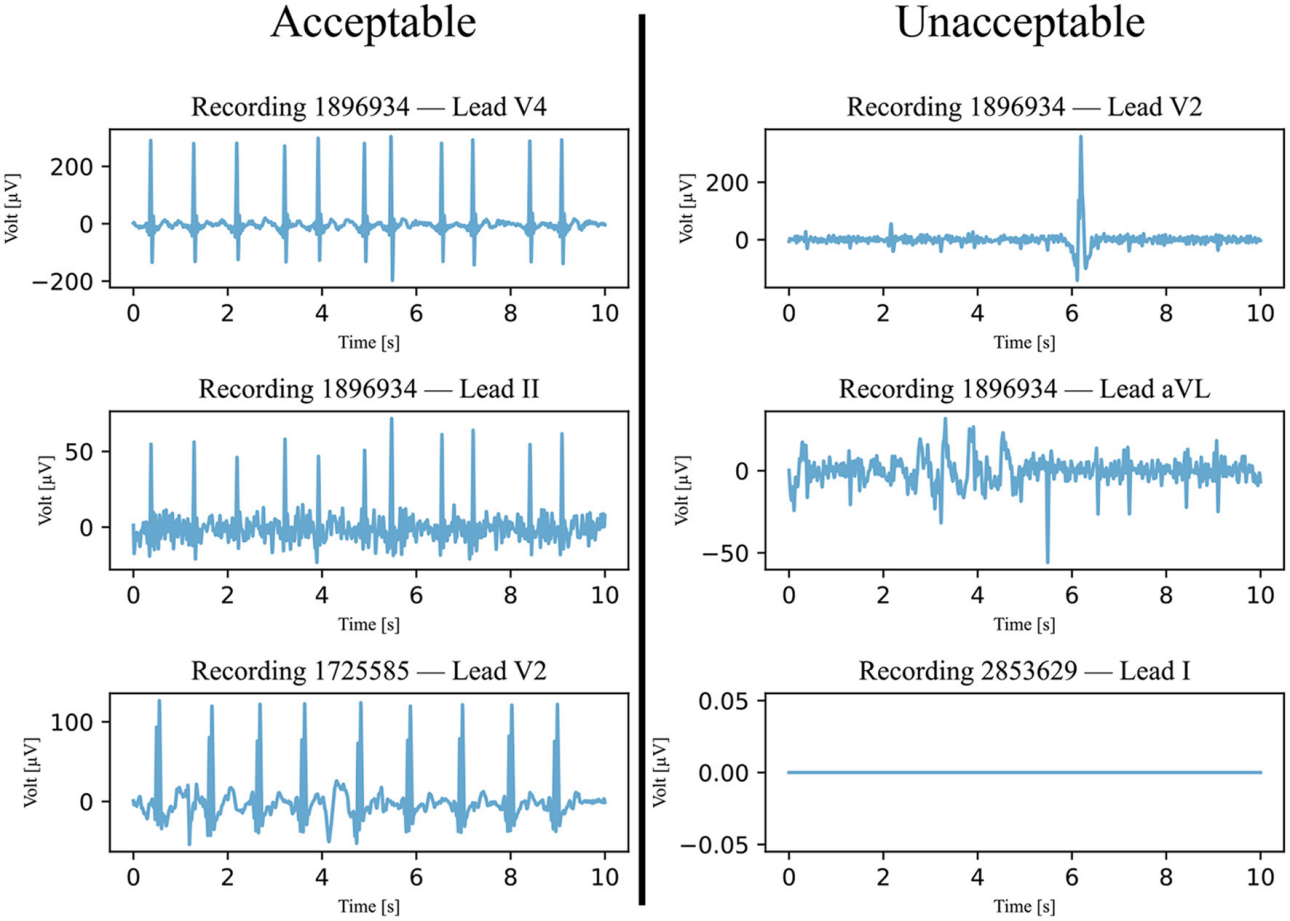
Six examples of ECG signals that were classified and annotated into acceptable and unacceptable with respect to their quality. The filtered signals were visually classified, with a focus on the heartbeats. If all heartbeats were visible, the quality was considered acceptable.

After preprocessing, the signals in the testing data set were categorized as acceptable or unacceptable. For the signal to be acceptable, all but the first and last heartbeats had to be visually identifiable. An example of this classification is shown in Figure 2. It is important to note that signals with a significant level of noise were accepted as well, but only if the heartbeats could be distinguished from the noise. Any signals that did not meet this condition were not accepted. This classification was separately completed by two annotators and disagreements were discussed and mutually decided upon.

### 2.3. Three Signal Quality Classification Algorithms

**Figure 3:**
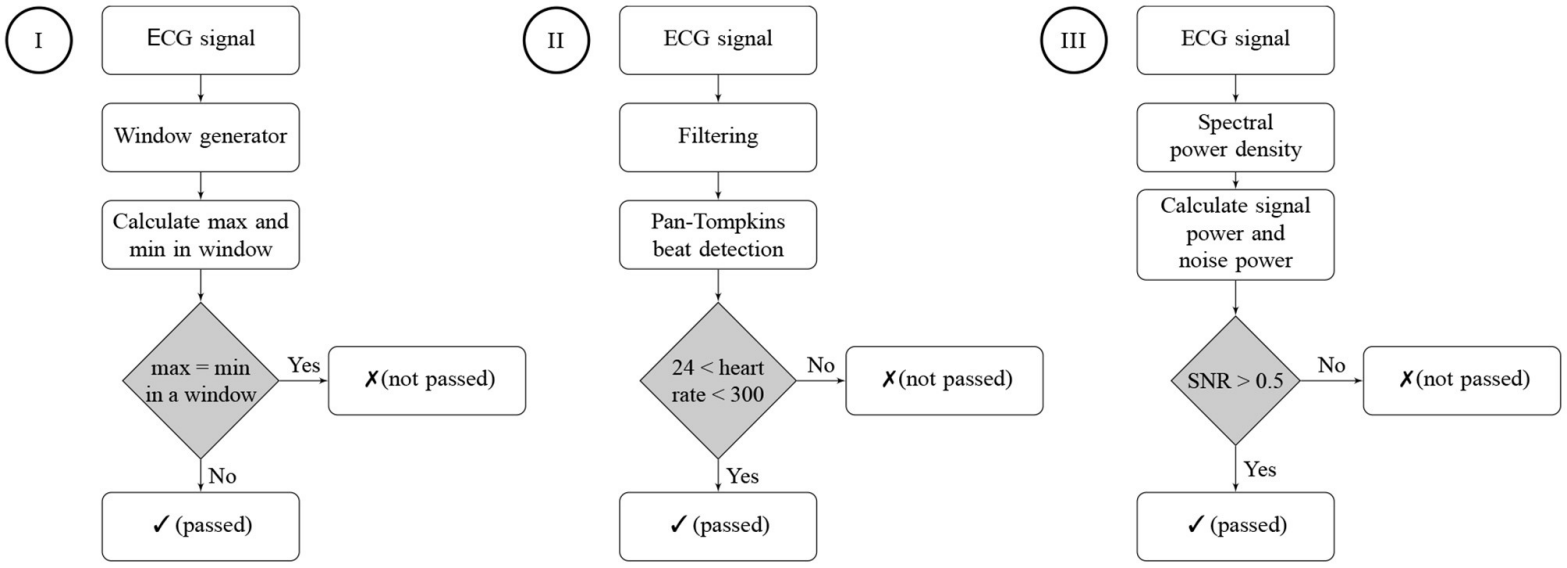
Flowcharts of the three signal quality classification algorithms. Algorithm I is a stationary check, Algorithm II is a heart rate check, and Algorithm III is an SNR check. A passed status on an algorithm means that no characteristics of a low-quality signal were detected. A not-passed status means that characteristics of poor quality were detected for the ECG signal.

The developed ECGAssess toolbox contains three algorithms that check the signals to determine their quality. Each of the three algorithms assigns a status of passed or not passed to each signal. The flowcharts of all three algorithms are shown in Figure 3.

#### 2.3.1. Algorithm I : Stationary Signal Check

**Figure 4:**
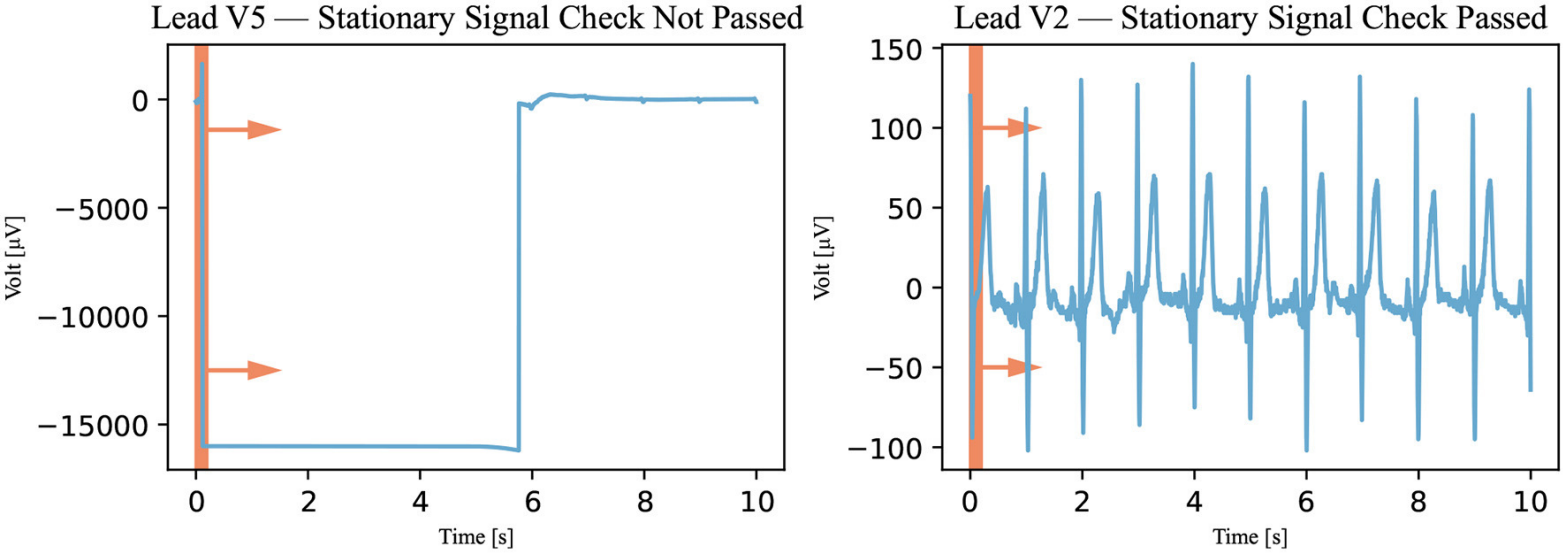
Stationary signal check. The window, represented by the red rectangle, moved through the signal (Recording 1072620) in the direction of the arrows, and the signal was viewed through the window in this algorithm. If the signal was stationary in the window at a specific time, it was declared not passed. A not-passed signal is shown on the left and a signal that has passed is shown on the right.

The first classification algorithm checks the signal on stretches where the signal remains stationary at one value, called either a *stationary signal* or a *flatline*. The signal was viewed and analyzed through a 0.2-s window (100 measurements). This window was technically realized by temporarily copying a section of the signal. These copies are 0.2-s long (100 measurements) and made at 0.02-s intervals (10-measurement). In all copies, the maximum and minimum values of the signals were calculated. If the maximum and minimum values of the same time window were equal, the signal was stationary and did not pass the assessment of this algorithm as demonstrated in Figure 4. It must be noted that only one window of 0.2 s (or 100 measurements) must be stationary for the signal to be declared not passed by the stationary signal detection algorithm.

#### 2.3.2. Algorithm II : Heart Rate Check

**Figure 5:**
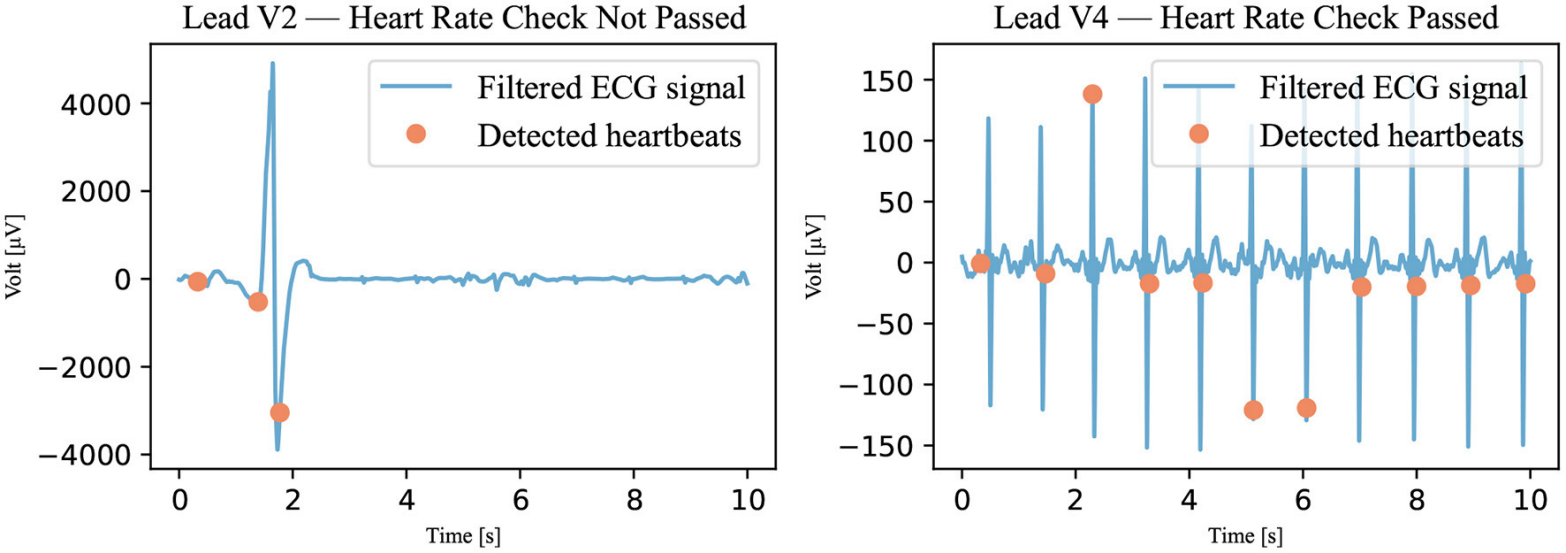
Heart rate check. The filtered signal (Recording 2167341) was analyzed using the Pan-Tompkins algorithm. The detected heartbeats are visible as dots. If the number of detected heartbeats was not within the accepted range, the signal was marked not passed. A signal that has not passed is shown on the left; a signal that has passed is shown on the right.

In this algorithm, the Pan-Tompkins algorithm was used to detect the QRS complex of the heartbeat (12). This algorithm was used because it is considered to be the gold standard for detecting R peaks (13). First, however, the signal had to be filtered. The same filter was used as for the annotation—that is, an 8–20 Hz bandpass filter (11). This prepared signal was analyzed with the Pan-Tompkins algorithm, and the detected heartbeats were counted. The heart rate was calculated by multiplying the number of detected beats by 6 (because the recordings only last for 10 s). If the heart rate was between 24 and 300 bpm, the signal was declared passed; otherwise, it was declared not passed as illustrated in Figure 5. The upper threshold of 300 bpm was chosen because a heart rate above this threshold is unsustainable (14). No literature was found for the lower threshold of awake patients, but an expert estimated it to be approximately 25 bpm.

#### 2.3.3. Algorithm III : Signal-to-Noise Ratio Check

The last classification algorithm checks how much noise is contained in the ECG signal. To express this in numbers, the signal-noise-ratio (SNR) is calculated. This quality index is defined as the ratio between the spectral power of the signal and that of the noise. A periodogram is used to approximate the power spectral density (PSD). After summing the power of the frequency bands of signal and noise, the SNR can be calculated with Formula 1.


(1) $$\begin{array}{l} \text{SNR=}\sum_{\text{2Hz}}^{\text{40Hz}}\text{P}\text{SD/}\sum_{\text{0Hz}}^{\text{2Hz}}\text{P}\text{SD +}\sum_{\text{40Hz}}^{\text{250Hz}}\text{P}\text{SD} \end{array}$$

The signal consists of P, Q, R, S, and T waves. Thus, the frequency range of the signal can be determined with the frequency ranges of these individual waves. The QRS complex has a frequency range of 8-50 Hz. The T wave and the P wave have frequency ranges of 0–10 and 5–30 Hz, respectively (15). Baseline drift is a low-frequency artifact in ECG signals and belongs to the noise spectrum. Baseline drift ranges from 0 to 2 Hz and is usually removed from the ECG signals before analysis (16), and studies have shown that a high frequency cutoff of 40 Hz enhances signal quality (17, 18). This leads to a signal frequency range of 2–40 Hz. The remaining frequencies are interpreted as noise. Noise mainly includes baseline drift, powerline interference, motion artifacts, and electromyography noise.

**Figure 6:**
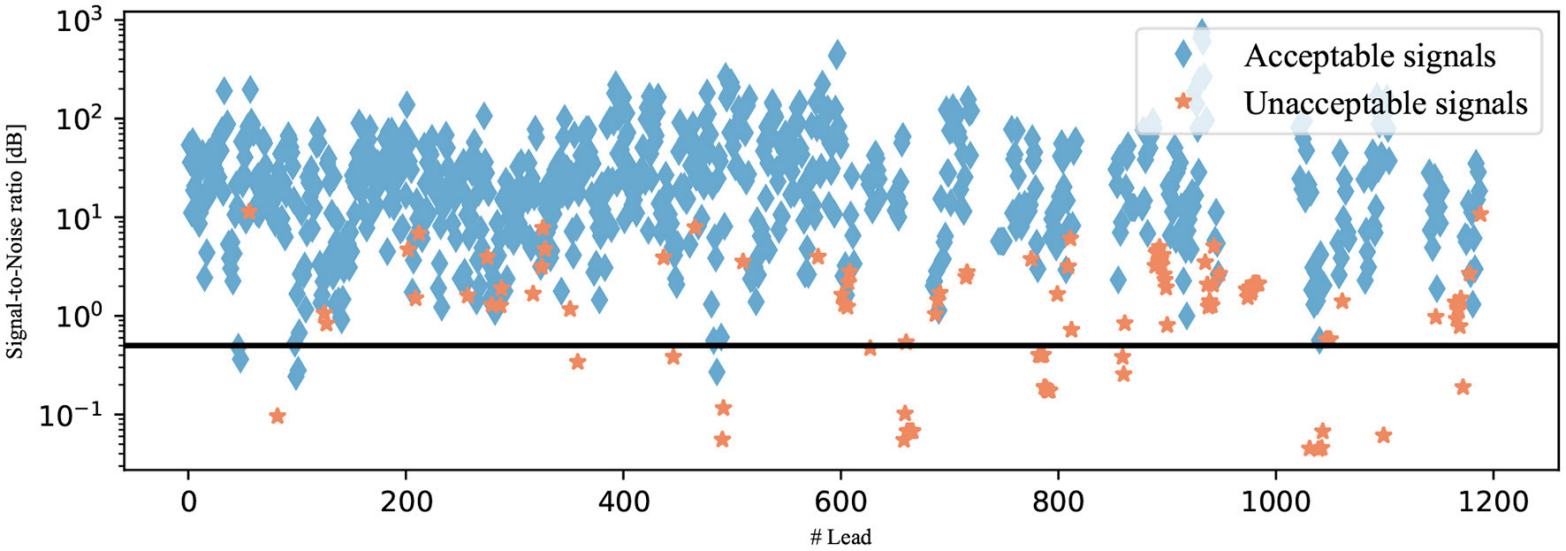
SNRs of the signals from the testing data set. The signals are shown as diamonds if they were annotated as acceptable and as stars if they were annotated as unacceptable. The selected SNR threshold of 0.5 dB is shown as a black line.

**Figure 7:**
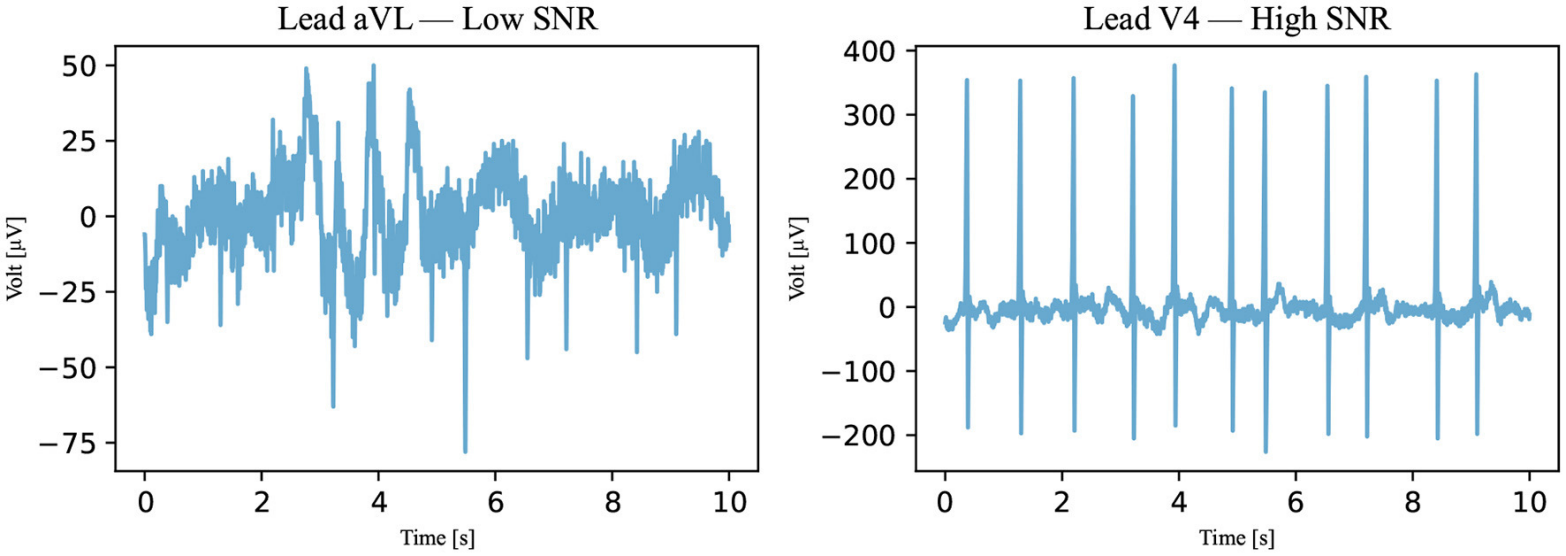
An SNR example. With respect to Algorithm III, an example of a not-passed signal is on the left and an example of a passed signal is on the right (Recording 1896934). The SNRs of the signals are 0.4384 dB for the signal on the left and 8.1156 dB for the signal on the right.

To identify a reasonable threshold value, the training data set is used. The SNRs of all annotated signals in this data set were calculated and are plotted in Figure 6 regarding the annotation of the signal. The different groups are shown to be partially separated from one another. Visually, the SNR threshold value of 0.5 dB is selected. The signals did not pass this last classification algorithm if their SNRs were below the selected threshold as illustrated in Figure 7.

### 2.4. Agreement Rule

To incorporate the advantages of all algorithms into the result, the algorithms were combined with an agreement rule. The following explanation is based on the example presented on Table 1. For the quality of a signal to be accepted, all three algorithms must agree on its good quality, as shown for Lead I. If a signal does not pass one or more of the algorithms, the quality of the signal is not accepted. All scenarios for unacceptable signals can be found in Lead II through V2.

**Table 1:** An example of the agreement rule for assessing the ECG quality of each lead.

**Leads**	**I**	**II**	**III**	**aVF**	**aVR**	**aVL**	**V1**	**V2**	**V3**	**V4**	**V5**	**V6**
Algorithm I	✓	✗	✗	✗	✓	✓	✓	✗	✓	✓	✓	✓
Algorithm II	✓	✓	✗	✗	✗	✗	✓	✓	✓	✓	✓	✓
Algorithm III	✓	✓	✓	✗	✗	✓	✗	✗	✓	✓	✓	✓
**Overall result**	✓	✗	✗	✗	✗	✗	✗	✗	✓	✓	✓	✓

*✓passed, ✗ not passed*.

## 3. Results and Discussion

ECG signal quality can be evaluated in different ways. A quality index can be assigned to the signal—for instance, from 0 to 1—or a binary model can be used, where a 0 or a 1 is assigned. In this study, a binary model, using acceptable and unacceptable statuses, was used because it allows for a distinct rule of assignment. An unambiguous criterion leads to little freedom in interpretation, resulting in a highly standardized method. Therefore, the process of assignment is simplified and made more transparent. Another reason for choosing a binary model is that it gives the toolbox's user clear feedback regarding whether a signal must be rerecorded.

During annotation, a signal was considered acceptable if a reliable heart rate could be detected; otherwise, the signal was considered unacceptable. The exact procedure is explained in the Method section. The results of this annotation for the testing data set are shown in Table 2.

**Table 2:** Results of the annotation and the total number of leads in the training and testing data sets.

	**Acceptable signals**	**Unacceptable signals**	**Total signals**
Training data set	815 (67.92%)	385 (32.08%)	1,200 (100%)
Testing data set	2,844 (67.71%)	1,356 (32.29%)	4,200 (100%)

The training data set was used to develop and optimize the algorithms. However, in Algorithm I and Algorithm II, however, this data set had no direct influence because data-independent criteria were used for the quality analysis. In contrast, in Algorithm III, the data from the training data set influenced the SNR threshold. The training data set results of the algorithms can be seen in Table 3.

**Table 3:** Results of the algorithms with respect to the training data set.

	**Algorithm I**	**Algorithm II**	**Algorithm III**	**Overall result**
True positive	815	815	798	798
False positive	75	295	335	44
True negative	310	90	50	341
False negative	0	0	17	17
**Correctly categorized**	93.75 %	75.42 %	70.67 %	**94.92 %**

In the end, the toolbox's functionality was determined with the use of the testing data set; this large data set was created for this sole purpose. Algorithms I, II, and III were separately tested and then tested in combination with the agreement rule. The results of the toolbox were compared to those of the annotation. It was found that 94.21% of the signals were correctly categorized as either acceptable or unacceptable. This result, as well as the separate results of the individual algorithms, are shown in Table 4. With respect to the testing data set, a sensitivity [TP/(TP+FN)] of 98.03% and a specificity [TN/(TN+FP)] of 86.21% were achieved.

**Table 4:** Results of the algorithms with respect to the testing data set.

	**Algorithm I**	**Algorithm II**	**Algorithm III**	**Overall result**
True positive	2,844	2,844	2,788	2,788
False positive	457	1,130	941	187
True negative	899	226	415	1,169
False negative	0	0	56	56
**Correctly categorized**	89.12 %	73.10 %	76.26 %	**94.21 %**

We checked whether the classification of the individual leads into acceptable and unacceptable statuses allows a conclusion to be drawn about the entire 12-lead ECG recording. The number of unacceptably annotated signals was identified for Group I and Group II, and it was found that the two groups could not be separated based on the number of unacceptable leads. This analysis was done with the testing data set. Therefore, no criteria can be established that allow the classification of the entire recording based on the quality of each individual lead.

The limitation of this study is the approach of annotation using a binary system. Classification by heart rate is an obvious approach, but a relatively large amount of information loss in the signals may be considered acceptable and may prevent a proper diagnosis. A next step would be to employ a quality index ranging from 0 to 1, although the assignment of such a process is considerably more complex and requires the expertise of cardiologists.

**Figure 8:**
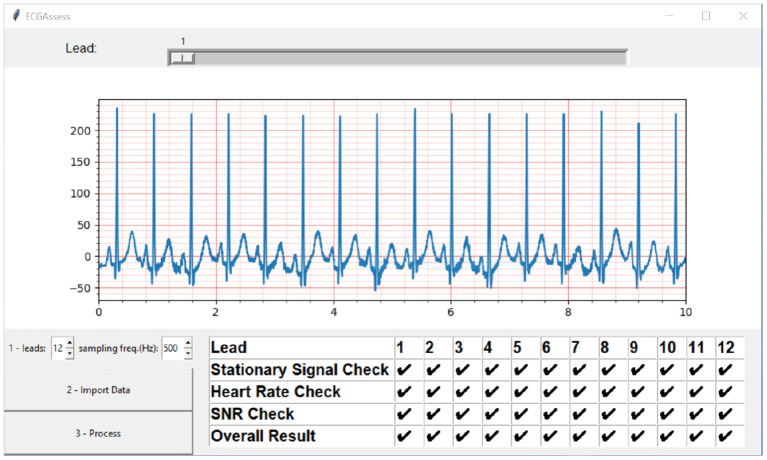
ECGAssess toolbox output. The toolbox graphical user interface was developed to enable people without programming knowledge to use the application. Pressing the Import Data button opens an explorer window where the .txt file of interest can be selected. The signal is also visible in the plot window with the standard ECG grid, to improve the interpretation to cardiologists. The visible lead can be selected using the slider. When the “Process” button is pressed, the signal quality assessment is executed, and the results are displayed in a table format.

The ECGAssess toolbox was developed as a dynamic model. New algorithms that analyze specific characteristics for poor signal quality can be quickly implemented and immediately tested in combination with the already developed building blocks. The toolbox was developed in Python for unrestricted access. Free PyCharm software was used as the integrated development environment. When necessary, the researchers used functions from public libraries, including NumPy, SciPy, ecg-detectors, Tkinter, and Matplotlib. To convert the code into an executable (.exe) file, the project auto-py-to-exe was used. The graphical user interface shown in Figure 8 was developed to enable people without programming knowledge to use the toolbox. The toolbox currently supports different types of ECG formats, such as .txt, .csv, .xls, .xlsx, and .wfdb, and it is worth noting that the program automatically recognizes the ECG file extension when it gets uploaded. The program can work with all sampling frequencies.

To examine the applicability of the toolbox in real-time, we calculated the order of complexity (O) for our algorithm. The calculations showed that O is linearly dependent on the number of ECG recordings, and estimated to be O (|*k*|*n), where *n* = number of ECG recordings and *k* = number of leads. We also ran the algorithm over an ordinary laptop [Dell XPS 13 9300, Intel(®) Core(™) i7-1065G7 CPU @ 1.30 GHz 1.50 GHz, 16.0 GB] and it takes 80 ms to process a 12-lead ECG recording.

## 4. Future Work

The authors want to encourage numbers of the scientific community to try their own algorithms with the toolbox and to find a good combination with already existing algorithms. The algorithms proposed in this project are by no means complete. Previously published algorithms can also be incorporated into the program and compared with the algorithms developed in this project (19, 20). To improve the adaptability and extensibility of the ECGAssess toolbox, an open API can be integrated in the future, suitable algorithms can be found on the internet.^1^

This is the first version (v1) of the ECGAssess toolbox that is currently trained over one data set. Additional data sets can be used to confirm the results. More ECG extension format, beat detectors (21, 22), signal quality assessment algorithms could be added. One of the next steps is to train the toolbox over different ECG data sets. We encourage members of the scientific community to download the toolbox and try the algorithms on their own data. In this article, we examined the binary classification (acceptable vs. unacceptable) of ECG signals, and one of the next steps is to investigate multi-classification (class 1 vs. class 2 vs. class 3). One of the databases is publicly available and contains three different types of ECG is the Brno University of Technology ECG Quality Database.^2^

## 5. Conclusion

Three algorithms—a stationary check, a heart rate check, and an SNR check—were used to classify the quality of ECG signals as either acceptable or unacceptable. The binary system provides a simple and unambiguous way to classify signals. The developed algorithms are uncomplicated in their realization; thus, they can be used in mobile devices with limited processing power. The feedback is instantaneously provided to the user. This study demonstrated a promising way to perform classification of ECG signal quality. Future versions of this ECGAssess toolbox may include instructions for proper placement of electrodes and detection and correction of incorrectly placed electrodes.

## Data Availability Statement

The original contributions presented in the study are included in the article/supplementary material, further inquiries can be directed to the corresponding author/s.

## Author Contributions

ME designed and led the study. LK, CM, and ME conceived the study. All authors approved final manuscript.

## Conflict of Interest

The authors declare that the research was conducted in the absence of any commercial or financial relationships that could be construed as a potential conflict of interest.

## Publisher's Note

All claims expressed in this article are solely those of the authors and do not necessarily represent those of their affiliated organizations, or those of the publisher, the editors and the reviewers. Any product that may be evaluated in this article, or claim that may be made by its manufacturer, is not guaranteed or endorsed by the publisher.

## Footnotes

^1^ https://github.com/cdepman/python-ECG-API

^2^ https://physionet.org/content/butqdb/1.0.0/
